# A Rare Case of Myocarditis in the Context of Norovirus Gastroenteritis

**DOI:** 10.1155/cric/5867611

**Published:** 2026-04-08

**Authors:** Eva S. Blake, Zachary T. St. Clair, Kent S. Brummel

**Affiliations:** ^1^ University of Michigan Medical School, Ann Arbor, Michigan, USA, umich.edu; ^2^ Department of Internal Medicine, Division of Hospital Medicine, University of Cincinnati, Cincinnati, Ohio, USA, uc.edu; ^3^ Department of Internal Medicine, Division of Cardiovascular Medicine, University of Michigan Health, Ann Arbor, Michigan, USA, uofmhealth.org

## Abstract

We present a rare case of viral myocarditis secondary to a rarely reported cause, norovirus. This case highlights the diagnostic challenges associated with acute chest pain accompanied by troponin elevation. We discuss the significance of advanced imaging techniques, particularly cardiac magnetic resonance, in the diagnosis of this condition.

## 1. Introduction

Although mostly associated with enteroviruses and, more recently, coronavirus‐19, viral myocarditis can be secondary to a wide variety of pathogens. For cases caused by atypical infections, the presentation may be difficult to distinguish from other causes of acute chest pain. The initial evaluation for chest pain must rule out obstructive coronary artery disease (CAD). Once completed, alternative explanations for the patient′s chest pain, such as stress cardiomyopathy, vasospastic angina, undifferentiated cases of myocardial infarction with nonobstructive coronary arteries (MINOCA), and viral myocarditis, should be pursued. Cardiac magnetic resonance (CMR) can be a useful and noninvasive tool in distinguishing etiology, particularly in cases with clinical ambiguity. We present a case of a 48‐year‐old woman who was diagnosed with acute viral myocarditis using CMR findings in the setting of an infection with an atypical myocarditis pathogen, norovirus.

## 2. Case Presentation

A previously healthy 48‐year‐old woman presented to the emergency department with left‐sided subjective weakness and numbness which was associated with chest pain radiating to the jaw. The patient also endorsed exertional dyspnea and noted a recent history of diarrhea, subjective chills, fevers, and headaches in the prior week. Regarding her family medical history, her father died from a myocardial infarction in his 70s. On initial presentation, her vital signs were stable and physical exam was largely unremarkable. Cardiac exam revealed normal S1 and S2 with no appreciable rubs, murmurs, or gallops, and the patient had no signs of volume overload. Initial neurologic evaluation demonstrated decreased sensation in the left arm and leg with no associated weakness or facial asymmetry. A stroke code was initiated and imaging (including noncontrast computed tomography [CT], CT angiography of the head and neck, and magnetic resonance [MR] of the brain) did not show evidence of ischemia or hemorrhage. The Neurology team evaluated the patient and had very low suspicion for stroke. Within 6 h of her arrival, her chest pain and left sided numbness resolved.

After stroke was ruled out, our assessment focused on the patient′s exertional chest pain in the setting of recent infectious symptoms. Our differential included acute coronary syndrome (ACS), myocarditis secondary to viral illness, vasospastic angina, and stress cardiomyopathy.

**Figure 1:**
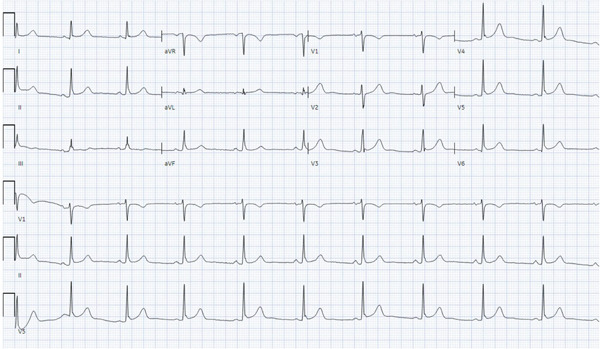
Initial electrocardiogram: no notable changes appreciated.

Laboratory evaluation revealed troponin elevation of 245 pg/mL (normal value < 19 pg/mL); repeat draw at 2 h showed further elevation to 375 pg/mL. Electrocardiogram (ECG) showed no significant abnormalities (see Figure 1). A basic metabolic panel was notable for a potassium of 3.0 mEq/L but otherwise all values were within normal limits. A lipid panel showed mildly elevated non‐HDL cholesterol at 140 mg/dL. Due to her symptoms and elevated troponin, the patient was started on heparin and given aspirin 325 mg, with a plan for coronary artery catheterization the next day. Echocardiogram revealed normal systolic function with no wall motion abnormalities and an ejection fraction of 58%.

**Figure 2:**
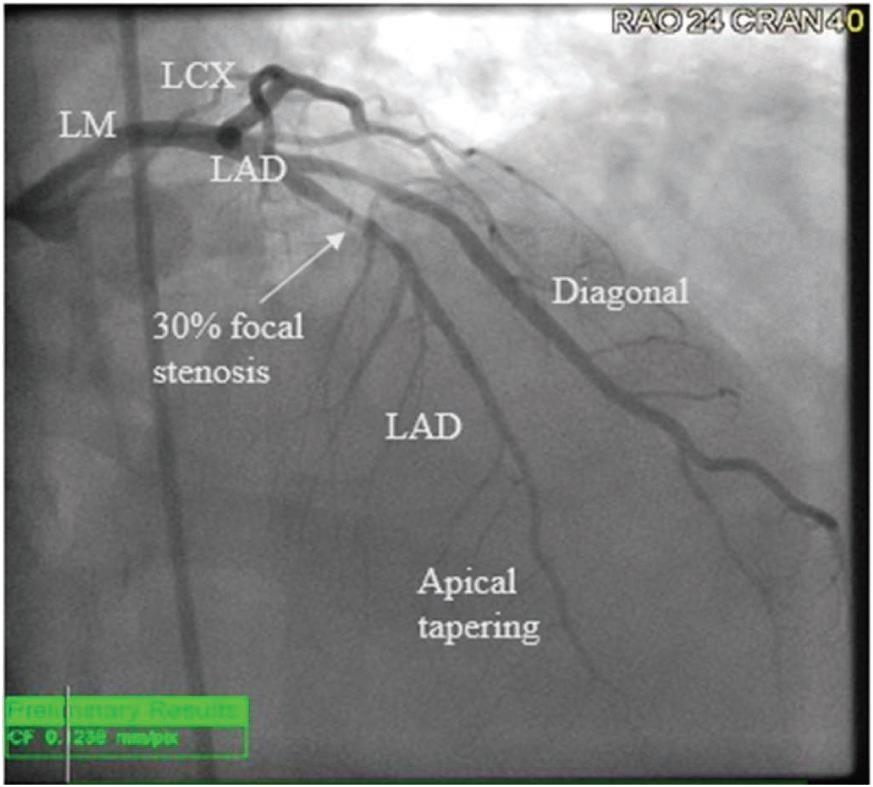
Catheterization: right anterior oblique cranial view demonstrating 30% focal stenosis in the LAD and apical tapering of the vessel.

**Figure 3:**
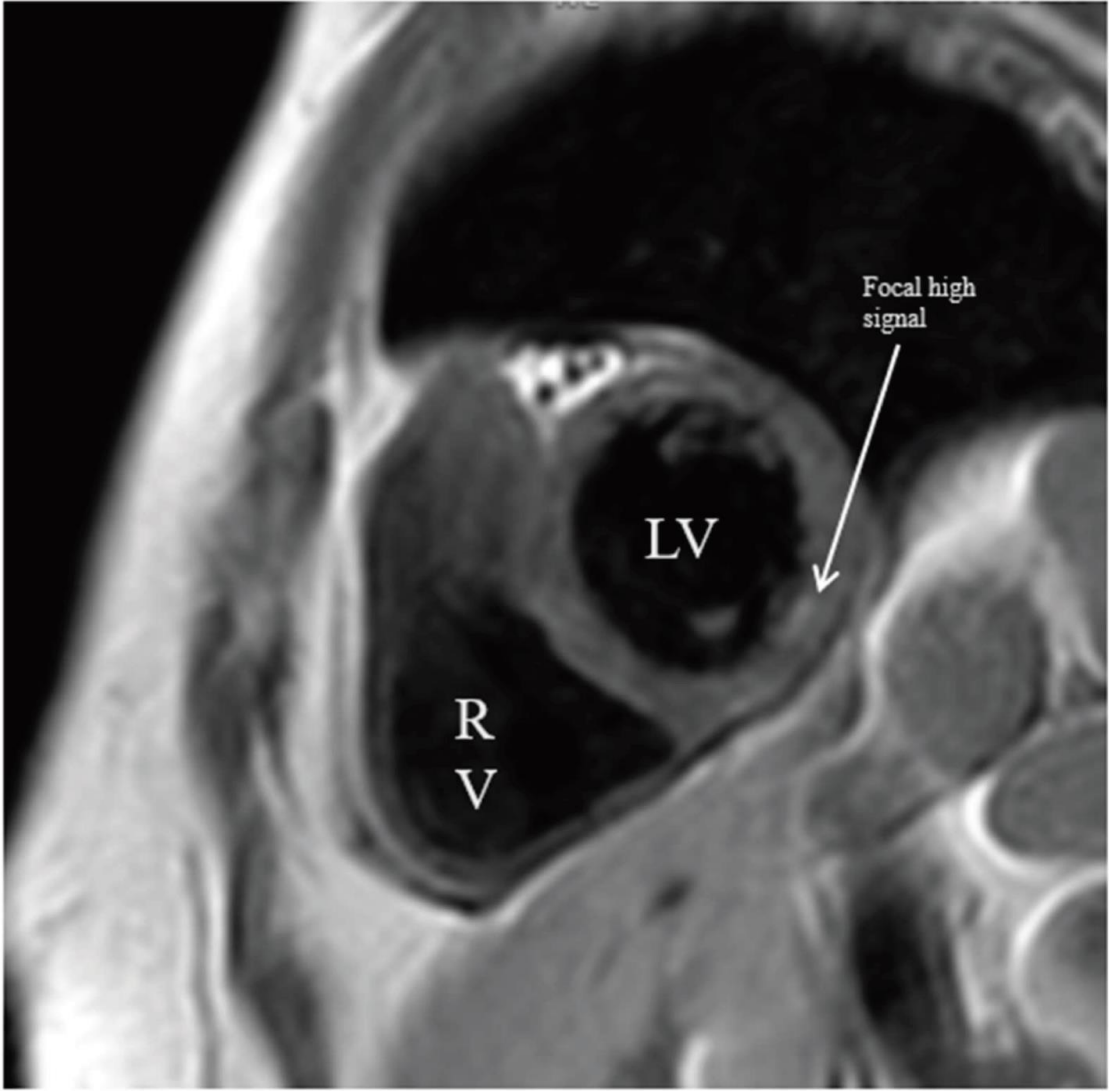
CMR T2‐weighted short‐axis images: midventricular hyperintensity in the inferolateral region corresponding to findings on LGE. Hyperintensity shown here is in the subendocardial region, typically associated with ischemia, although not in a vascular territory.

**Figure 4:**
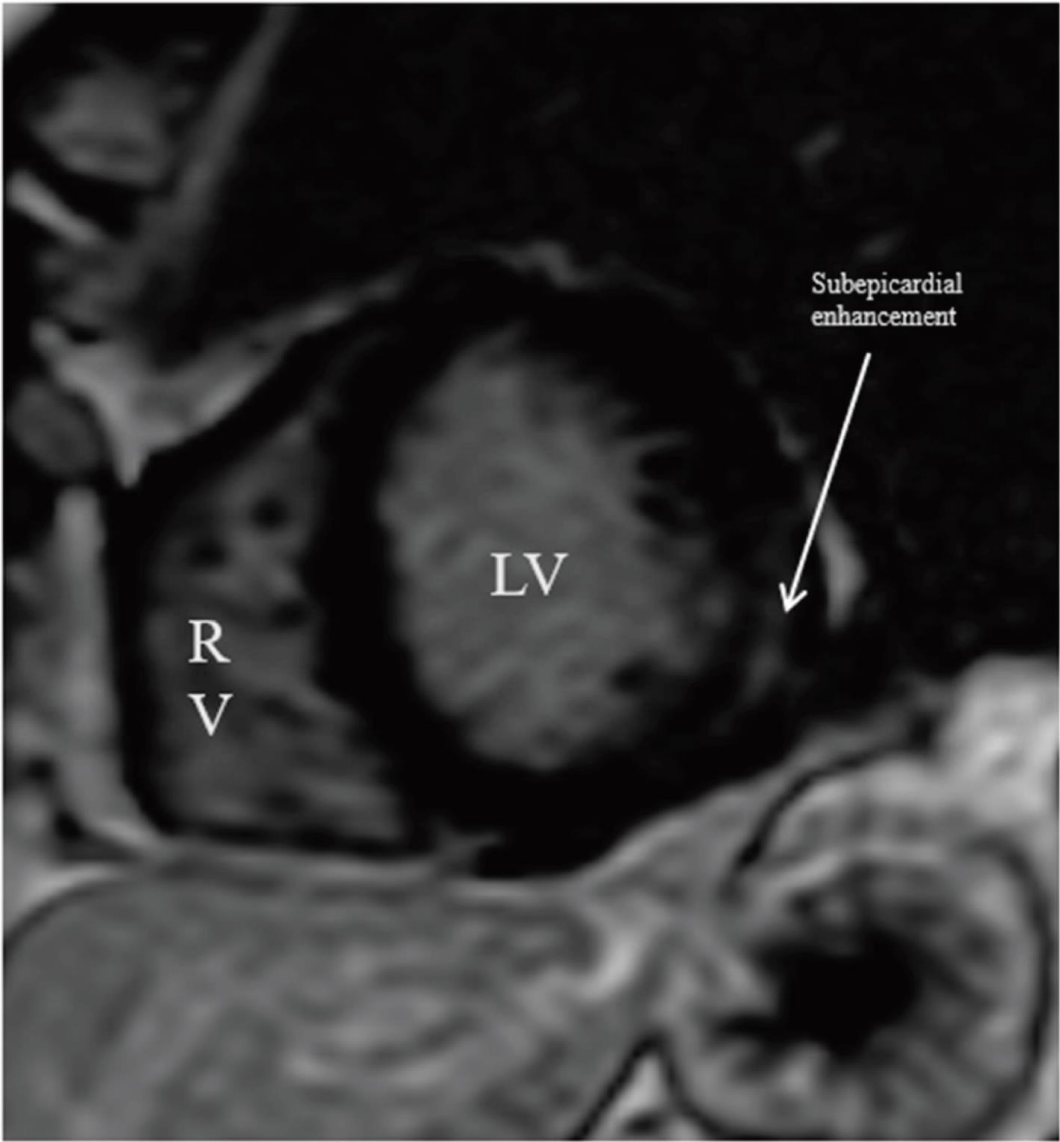
CMR LGE short‐axis images: delayed subepicardial enhancement seen in the inferolateral region corresponding to associated hyperintensity on T2 images, more consistent with myocarditis.

**Figure 5:**
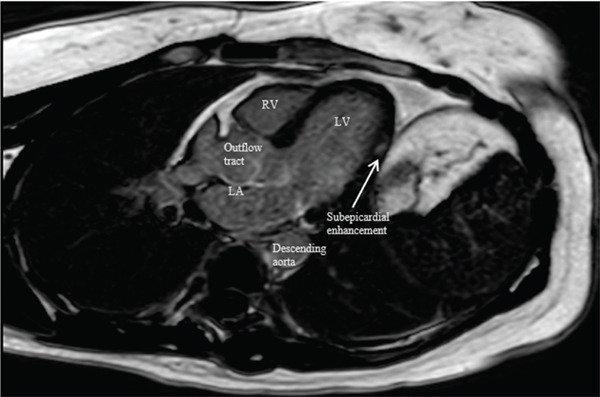
CMR LGE three‐chamber images: delayed enhancement redemonstrated in subepicardial midventricular wall.

Catheterization was significant for 30% stenosis in the left anterior descending artery which was noted to be apically diminutive and there was an additional nonobstructive lesion in the second diagonal branch (see Figure 2). A gastrointestinal pathogen panel, which tested for 21 unique pathogens, returned positive for norovirus, confirming acute norovirus gastroenteritis. Other negative infectious testing included a respiratory pathogen panel and a noninflammatory urinalysis, decreasing the likelihood of coinfection. Given the lack of a clear culprit lesion on angiography, further work‐up was pursued with CMR. CMR demonstrated focal high signal on T2‐weighted imaging involving the mid ventricular inferolateral wall (see Figure 3) with accompanying delayed enhancement seen subepicardially in the short axis and three chamber orientations (see Figures 4 and 5). These findings were consistent with a diagnosis of clinically suspected myocarditis secondary to norovirus infection.

The patient was discharged with counseling to restrict alcohol and physical activity until further follow‐up with her cardiologist. Given her normal cardiac function, she was not started on guideline directed medication for heart failure. For her concurrent finding of nonobstructive CAD, she was prescribed 80 mg of atorvastatin daily and 81 mg of aspirin daily. At an appointment 8 weeks after initial presentation, the patient did not report any new episodes of chest pain, dyspnea, paroxysmal nocturnal dyspnea, syncope, or palpitations. A follow‐up transthoracic echocardiogram is planned at 6 months for interval follow‐up.

## 3. Discussion

This case illustrates the diagnostic challenge of patients presenting with MINOCA. Of note, there is current debate over whether myocarditis should be included under the umbrella diagnosis of MINOCA, but we refer to them here as two distinct entities for clarity. Although this patient′s clinical history pointed towards the final diagnosis of acute viral myocarditis, possible alternative etiologies included microvascular atherosclerosis or coronary vasospasm. Although her gastrointestinal symptoms supported myocarditis, other cardiac conditions are likewise more prevalent in the setting of an acute illness and with physical and emotional stress [1]. With angiography demonstrating only minimally obstructive CAD, microvascular dysfunction and coronary vasospasm were important considerations. In cases with signs of heart failure or functional changes, it can also be difficult to distinguish myocarditis from stress cardiomyopathy. CMR can be a useful tool in differentiating patients with a working diagnosis of MINOCA; a recent meta‐analysis demonstrated that 68% of patients with an initial MINOCA diagnosis were able to be reclassified, into such diagnoses as myocarditis or stress cardiomyopathy [2].

Current guidelines recommend a three‐tiered approach to the diagnosis of myocarditis: acute evaluation, confirmation of myocarditis diagnosis, and follow‐up. As our patient was clinically stable and there was low concern on acute evaluation for either fulminant or complicated myocarditis, CMR was indicated as a first‐line choice for diagnostic confirmation. Endomyocardial biopsy, the gold standard for diagnosis, is indicated in patients with cardiogenic shock, complicated myocarditis, myocarditis associated with eosinophilia, or those with chronic inflammatory cardiomyopathy [3].

CMR is a valuable tool in confirming a myocarditis diagnosis and differentiating from other potential etiologies. The 2018 Lake Louise criteria have been shown to have a particularly high sensitivity in those presenting with an infarct‐like presentation, like our patient [4]. Coronary artery structure, however, is poorly evaluated on CMR, and evaluation for coronary anatomy, atherosclerosis, or dissection is better conducted via invasive catheterization or coronary CT angiography. Both T1 and T2 sequences from CMR can be used to support a diagnosis of myocarditis, in addition to providing prognostic information [3]. Myocarditis is associated with subepicardial injury, often multifocal, whereas infarction (whether due to plaque rupture or SCAD) is associated with subendocardial (and can extend distally depending on the extent of injury) enhancement in a vascular territory. Stress cardiomyopathy may present with myocardial edema on CMR but is much less likely to show delayed enhancement [5].

In our patient, CMR demonstrated findings consistent with myocarditis, including subepicardial enhancement on LGE and associated focal high signal on the T2‐weighted imaging [6]. These findings, along with the patient′s clinical history and troponin elevations, supported a final diagnosis of myocarditis, albeit with an unusual suspected trigger of acute norovirus infection (Figure 6).

Figure 6Typical distributions of CMR findings in ischemic injury (a) and myocarditis (b).

**Figure:**
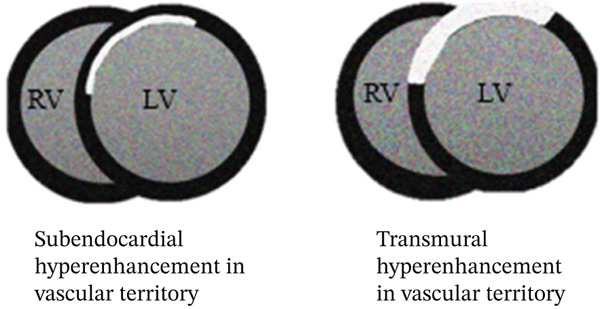
(a)

**Figure:**
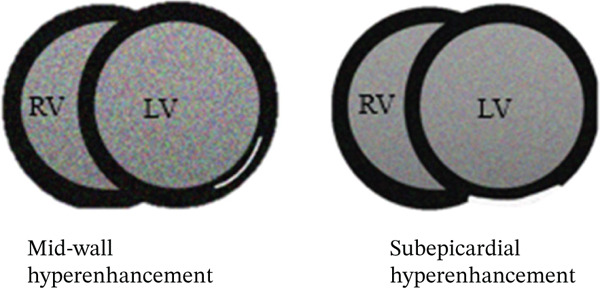
(b)

Viral infection is the most implicated cause of myocarditis in the United States and was the probable cause in our patient with fevers, chills, and acute diarrhea preceding her chest pain. Although enteroviruses are traditionally implicated in myocarditis, recent studies have demonstrated wide‐ranging viral causes [7]. At this time, 22 viruses have been demonstrated on myocardial biopsy, although a broader number of viruses are hypothesized to be potential causes. Norovirus, the suspected cause for our case, has only been reported once previously in the literature and that case was not confirmed by either endomyocardial biopsy or imaging [8]. Our case adds to a growing body of reports of atypical infectious myocarditis. Recent publications describe a case of biopsy confirmed thogotovirus‐associated myocarditis, a patient with suspected myocarditis in the setting of Salmonella gastroenteritis, and a man with CMR diagnosed myocarditis following recent erypiselas of the right foot [9–11]. These cases support the suspicion of recent literature that myocardial damage can arise from a number of pathologies, including viruses that were not previously known to be cardiotropic.

A variety of mechanisms for viral myocarditis have been proposed. On biopsy, viral myocarditis is associated with lymphocytic infiltrate, but cardiac damage may be secondary to a variety of pathologies. Direct viral invasion is associated with enteroviruses, endothelial dysfunction is a one of the suggested mechanisms of coronavirus‐19, and an immune response is thought to be the method of damage in influenza infection [12]. Regarding norovirus′s potential mechanism of systemic toxicity, some research has suggested that the virus binds to human histo‐blood group antigens, thus activating an immune response and causing systemic effects [8]. Lymphocytic infiltrates may not be virally mediated; other common etiologies include immune checkpoint inhibitors and systemic autoimmune diseases. Clinical history must guide diagnosis to distinguish between these causes.

Appropriate treatment for viral myocarditis seems to hinge upon the underlying pathophysiology. For lymphocytic myocarditis, the presence of high viral loads on endomyocardial biopsy has been suggested as an indication for antiviral treatment and a contraindication for immunosuppressive therapies. There has not been robust data establishing either as standard of care for acute lymphocytic myocarditis, however, and, as in our patient, endomyocardial biopsy is often not indicated without more specific indications. Promising studies have shown the role of antiviral and immunosuppressive therapies in chronic inflammatory cardiomyopathy, but likewise are not part of current American Heart Association guidelines [12]. In comparison to lymphocytic myocarditis, treatment recommendations for eosinophilic and giant cell myocarditis, respectively, consist of a course of corticosteroids and immunosuppressive therapy. Thus, for patients with clinical findings suspicious of an eosinophilic etiology or those with significant complications as discussed previously, endomyocardial biopsy is recommended to help guide targeted treatment [12].

In addition to targeted treatment, general management principles for heart failure and arrhythmia therapy should be employed. In our patient, given the absence of heart failure or persistent symptoms, treatment recommendations focused on alcohol and exercise restriction for 3–6 months [13]. Interval follow‐up for this low‐risk patient includes echocardiography to reassess for the development of late‐onset heart failure [3].

## 4. Conclusion

We present a case of acute chest pain and nonobstructive CAD, found to be myocarditis in the setting of a norovirus infection. This case highlights the important role of imaging in differentiating myocarditis from other causes of acute chest pain.

## Funding

No funding was received for this manuscript.

## Disclosure

The authors have nothing to report.

## Ethics Statement

Informed consent was secured from the patient for all treatments and procedures.

## Consent

No written consent has been obtained from the patients as there is no patient identifiable data included in this case report.

## Conflicts of Interest

The authors declare no conflicts of interest.

## Data Availability

The data that support the findings of this study are available on request from the corresponding author. The data are not publicly available due to privacy or ethical restrictions.
